# Intermediate hepatitis B virus infection prevalence among 1622 pregnant women in rural Burkina Faso and implications for mother-to-child transmission

**DOI:** 10.1038/s41598-023-32766-3

**Published:** 2023-04-14

**Authors:** Serge Ouoba, Ko Ko, Moussa Lingani, Shintaro Nagashima, Alice N. Guingané, E. Bunthen, Md Razeen Ashraf Hussain, Aya Sugiyama, Tomoyuki Akita, Masayuki Ohisa, Moussa Abdel Sanou, Ousmane Traore, Job Wilfried Nassa, Maimouna Sanou, Kazuaki Takahashi, Halidou Tinto, Junko Tanaka

**Affiliations:** 1 Department of Epidemiology, Infectious Disease Control and Prevention, Graduate School of Biomedical and Health Sciences, Hiroshima University, 1-2-3, Kasumi, Minami-Ku, Hiroshima, 734-8551 Japan; 2 Unité de Recherche Clinique de Nanoro (URCN), Institut de Recherche en Science de la Santé (IRSS), Nanoro, Burkina Faso; 3 Unite de Formation Et de Recherche en Sciences de la Sante, Universite Joseph Ki-Zerbo, Ouagadougou, Burkina Faso; 4 Payment Certification Agency (PCA), Ministry of Health, Phnom Penh, Cambodia

**Keywords:** Epidemiology, Hepatitis B

## Abstract

In highly endemic countries for hepatitis B virus (HBV) infection, childhood infection, including mother-to-child transmission (MTCT), represents the primary transmission route. High maternal DNA level (viral load ≥ 200,000 IU/mL) is a significant factor for MTCT. We investigated the prevalence of HBsAg, HBeAg, and high HBV DNA among pregnant women in three hospitals in Burkina Faso and assessed the performance of HBeAg to predict high viral load. Consenting pregnant women were interviewed on their sociodemographic characteristics and tested for HBsAg by a rapid diagnostic test, and dried blood spot (DBS) samples were collected for laboratory analyses. Of the 1622 participants, HBsAg prevalence was 6.5% (95% CI, 5.4–7.8%). Among 102 HBsAg-positive pregnant women in DBS samples, HBeAg was positive in 22.6% (95% CI, 14.9–31.9%), and viral load was quantified in 94 cases, with 19.1% having HBV DNA ≥ 200,000 IU/mL. HBV genotypes were identified in 63 samples and predominant genotypes were E (58.7%) and A (36.5%). The sensitivity of HBeAg by using DBS samples to identify high viral load in the 94 cases was 55.6%, and the specificity was 86.8%. These findings highlight the need to implement routine HBV screening and effective MTCT risk assessment for all pregnant women in Burkina Faso to enable early interventions that can effectively reduce MTCT.

## Introduction

The burden of hepatitis B virus (HBV) infection disproportionally affects regions of the world. Of the 296 million people chronically infected with HBV worldwide in 2019, around 81 million people were living in Africa^1^. Moreover, Africa has the highest prevalence of HBV infection in children under five years, and 70% of new infections in the world occur in the African region^2^. Infection in childhood, including mother-to-child transmission (MTCT), represents the primary transmission route and progression to chronicity, estimated at 90% when infected before five years old^3^. MTCT can occur in-utero, at birth, or postnatally through close contact with an infected mother or via breastfeeding^4^.

The World Health Organization (WHO) aims to eliminate hepatitis B as a public health threat by 2030^5^. Achieving this goal requires reducing HBV prevalence to less than 0.1% in children under five^6^. Therefore, preventing mother-to-child transmission (PMTCT) of HBV is critical. WHO guidelines for the PMTCT of HBV are stratified into three pillars: (i) Administration of at least 3-dose HBV vaccine to newborns, including a timely birth dose, (ii) screening of pregnant women for HBsAg and administration of hepatitis B immunoglobulins (HBIG) (if available) to infants born to HBsAg-positive and HBeAg-positive mothers, (iii) antiviral prophylaxis in pregnant women with high HBV viral load (HBV DNA ≥ 200,000 IU/mL) or Hepatitis B e antigen (HBeAg) positive^7^.

The global elimination of HBV requires information on the characteristics of HBV infection among pregnant women, those at high risk of transmitting the virus to their offspring (i.e., those with high viral load or HBeAg positive), and the suitability of control measures across various settings.

Burkina Faso, a West African country, is highly endemic to HBV infection with an estimated nationwide prevalence of 9.1% in 2010^8^. Since 2006, the hepatitis B vaccine in childhood has been introduced as part of the Expanded Program on Immunization (EPI), but universal hepatitis B vaccination at birth is not yet available. Also, routine screening for pregnant women is not established. Transmission routes in adolescents and adult women include ritual scarifications, female genital mutilation (FGM), unsafe blood transfusion, and exposure to contaminated blood^9^.

In this study, we investigated the prevalence of HBsAg and markers of high risk for MTCT (HBeAg, HBV DNA) among pregnant women in rural Burkina Faso, and assessed the performance of HBeAg to predict high viral load.

## Methods

### Study setting and subjects selection

This study is part of a longitudinal research aiming to assess MTCT in Burkina Faso. Within this research, we conducted a cross-sectional study among pregnant women attending antenatal care. This study was conducted between February and November 2021 in three first-level public health centers in the Yako health district, a rural setting in Northern Burkina Faso. Details of the study setting are described elsewhere^10^.

All antenatal care attendees during the study period were invited to participate. A consecutive sampling method was applied, and consenting pregnant women were subjected to a questionnaire on their sociodemographic characteristics. HIV infection status was obtained from medical records. Hepatitis B surface Antigen (HBsAg) was tested using a highly sensitive rapid diagnostic point of care with an analytical sensitivity of 0.1 IU/mL (Determine HBsAg 2, Abbott Laboratories, IL, USA). Then, dried blood spot (DBS) samples were collected by finger prick from HBsAg-positive pregnant women using the Hemaspot™ device (Spot on Sciences, CA, USA). All DBS samples were stored at -20 degrees before shipment to the analytical laboratory at Hiroshima University, Japan, for serological and molecular analyses.

### Serological assays

Three fins of DBS samples were detached and eluted as previously described^11^, and analyzed on Lumipulse G1200 (Fujirebio Inc., Tokyo, Japan), a chemiluminescent enzyme immunoassay (CLEIA) automated analyzer. HBsAg, HBeAg, and HBeAb, were detected using Lumipulse® HBsAg-HQ, Lumipulse® HBeAg, and Lumipulse® HBeAb, respectively. The accuracy of DBS samples to detect HBV seromarkers was evaluated based on our previously published study that compared DBS samples to paired serum samples^12^. The reported sensitivities for HBsAg, HBeAg, and HBeAb were 89.3%, 100%, and 100%, respectively, with a 100% specificity for all seromarkers.

### HBV DNA quantification and genotype determination

One DBS fin was taken to extract HBV DNA using the SMITEST EX‐R&D kit (Medical & Biological Laboratories Co., Ltd., Japan) and perform DNA quantification and genotype identification.

HBV DNA was quantified by real-time polymerase chain reaction (PCR) using the TaqMan Fast Advanced Master Mix reagent (Thermo Fisher Scientific, MA, USA) on Applied Biosystems StepOne (Thermo Fisher Scientific, MA, USA).

Using PrimeScript One-Step RT-PCR Kit Ver.2, HBV DNA was amplified by nested PCR targeting the overlapping surface-polymerase (SP) or the surface region of the viral genome in case the SP region amplification failed. PCR products were then sequenced by the Sanger sequencing method on SeqStudio Sequence Analyzer (Thermo Fisher Scientific, MA, USA) using the BigDye Terminator v3.1 Cycle Sequencing Kit (Thermo Fisher Scientific, CA, USA). Then, HBV genotypes were identified by analyzing partial genome sequences with 103 reference strains retrieved from GenBank, using the neighbor-joining method with the Molecular Evolutionary Genetics Analysis software version 10 (Pennsylvania State University, PA, USA). The resulting phylogenetic trees are shown in Supplementary Figs. S1 and S2. Supplementary Table S1 shows the primers used for nested PCR reactions and genome sequencing^13,14^.

### Sample size calculation

The required sample size for the study was estimated using the following formula: $\mathrm{N}=\frac{{1.96}^{2}\times (\mathrm{p}\times (1-\mathrm{p}))}{{\mathrm{d}}^{2}}\times DE\times \frac{1}{R}$, where N = sample size, p = expected proportion, d = precision, DE = design effect, R = response rate. Considering an expected prevalence (p) of 8.7%^15^, a precision (d) of 2%, a design effect (DE) of 1.5, and a response rate (R) of 80%, the calculated minimum sample size was 1,431. We finally included 1622 pregnant women.

### Data management and analysis

Questionnaire data were collected on electronic tablets, encoded onto the Research Electronic Data Capture (RedCap, version 8.9.2), and transferred to the Clinical Research Unit of Nanoro server. Data were then exported to Stata 16.1 (StataCorp LLC, TX, USA, 2020) for data management and statistical analysis.

Categorical variables are presented as numbers and percentages, continuous variables as mean and standard deviation or median and interquartile range, as appropriate. The Chi-square test was used to compare categorical variables and the Wilcoxon rank-sum test for continuous variables.

The factors associated with HBsAg prevalence were analyzed by multivariable logistic regression with the following variables as cofactors: age group, history of transfusion, surgery, piercing, scarification, and FGM. Besides, the backward stepwise method was employed to select additional cofactors among occupation, education, parity, HIV infection, and having ever heard of HBV (probability of entry *p* < 0.25, probability of removal *p* < 0.3).

The level of significance of all statistical analyses was 0.05.

### Ethical considerations

The study received ethical approval from the Burkina Faso National Ethics Committee (approval number 2020-8-145) and the Ethics Committee for epidemiological research of Hiroshima University (approval number E-2137). The study was performed in conformity with the Declaration of Helsinki and regulations in Burkina Faso. Informed consent was obtained from all study participants before any study procedures. Pregnant women positive for HBV infection were followed according to the guidelines on the PMTCT of HBV in Burkina Faso.

## Results

### Study participants characteristics

**Figure 1:**
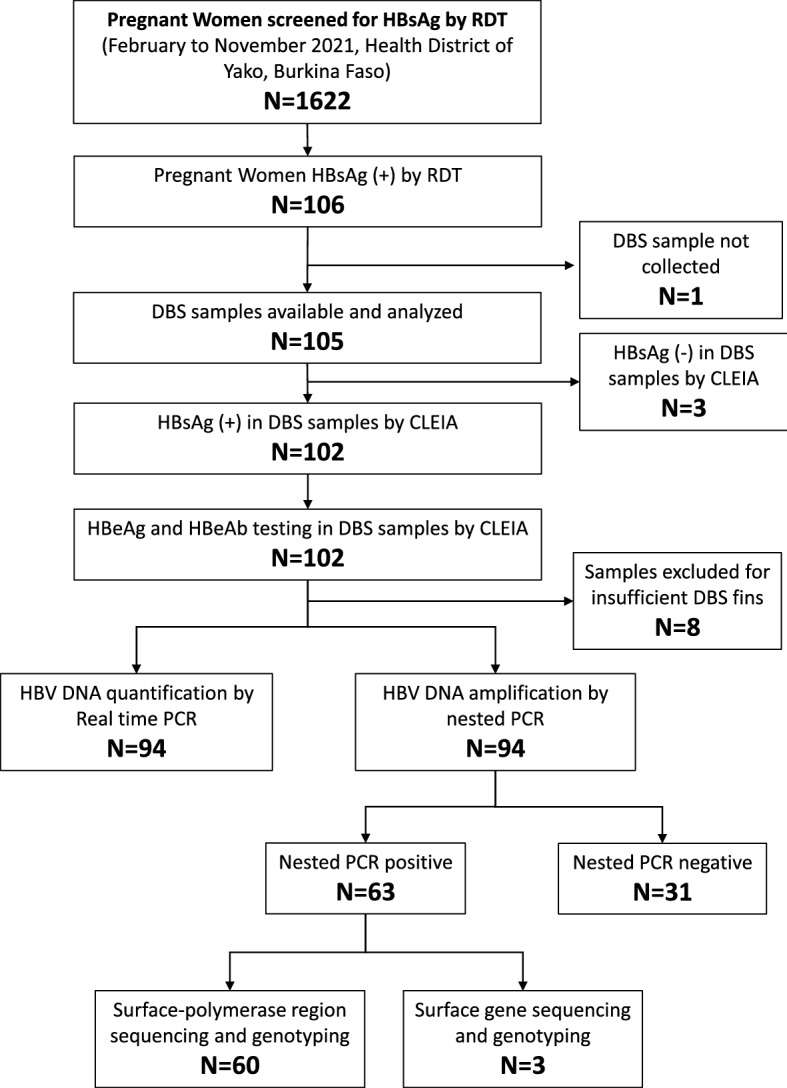
Study and laboratory analysis flow. RDT, Rapid diagnostic test; CLEIA, chemiluminescent enzyme immunoassay; HBsAg, Hepatitis B surface antigen; HBeAg, Hepatitis B e antigen; HBeAb, Hepatitis B e antibody, DBS, Dried Blood Spot.

Figure 1 shows the study and laboratory analysis flow. A total of 1622 pregnant women were enrolled in the study. The mean age was 25.1 ± 6.0 years (range 15–46 years). All pregnant women were born before 2006, when HBV vaccination was introduced in the Expanded Program on Immunization in children in Burkina Faso. Regarding risk factors for HBV infection, 1.6% received transfusion in the past, 3% underwent surgery, 80.0% had a history of piercing, 30.8% underwent ritual scarifications, and 73.1% had a history of FGM. The study subject characteristics are shown in Table 1.

**Table 1:** HBsAg prevalence and logistic regression analysis of factors associated with HBsAg among pregnant women in Yako, rural Burkina Faso.

Variable	Study population N (%)	HBsAg prevalence by RDT		Univariable logistic regression		Multivariable logistic regression	
		n	% (95% CI)	OR (95% CI)	*p*-value	aOR (95% CI)	*p*-value
Overall	1622 (100)	106	6.5 (5.4–7.8)	–	–	–	–
Age group (years)							
15–24	826 (50.9)	49	5.9 (4.4–7.8)	1		1	
25–34	596 (36.7)	45	7.6 (5.6–10.0)	1.30 (0.85–1.97)	0.227	2.24 (1.28–3.93)	0.005
35–46	146 (9.0)	11	7.5 (3.8–13.1)	1.29 (0.66–2.55)	0.460	2.15 (0.89–5.18)	0.089
Missing data	54 (3.3)	–		–	–	–	–
Occupation							
Housewife/farmer	1244 (76.7)	81	6.5 (5.3–8.0)	0.88 (0.47–1.64)	0.681		
Employee	163 (10.1)	12	7.4 (3.9–12.5)	1			
Student	80 (4.9)	5	6.3 (2.1–14.0)	0.84 (0.29–2.47)	0.750		
Missing data	135 (8.3)	–		–	–	–	–
Education							
No or non-formal	686 (42.3)	48	7.0 (5.2–9.2)	1.11 (0.69–1.78)	0.662		
Primary education	435 (26.8)	27	6.2 (4.1–8.9)	0.98 (0.57–1.67)	0.933		
Secondary or tertiary education	473 (29.2)	30	6.3 (4.3–8.9)	1			
Missing data	28 (1.7)	–		–	–	–	–
Parity							
Nulliparous (no previous birth)	414 (25.5)	29	7.0 (4.7–9.9)	1		1	
Primiparous (1 previous birth)	434 (26.8)	31	7.1 (4.9–10.0)	1.02 (0.6–1.73)	0.938	0.46 (0.23–0.91)	0.509
Multiparous (> 1 previous birth)	652 (40.2)	40	6.1 (4.4–8.3)	0.87 (0.53–1.42)	0.574	0.83 (0.47–1.45)	0.025
Missing data	122 (7.5)	–		–	–	–	–
Ever heard of HBV							
Yes	876 (54.0)	44	5.0 (3.7–6.9)	1		1	
No	724 (44.6)	61	8.4 (6.5–10.7)	1.74 (1.17–2.6)	0.007	1.63 (1.07–2.49)	0.022
Missing data	22 (1.4)	–		–	–	–	–
Transfusion							
Yes	25 (1.5)	2	8.0 (1.0–26.0)	1.25 (0.29–5.36)	0.768	1.43 (0.31–6.52)	0.646
No	1579 (97.4)	103	6.5 (5.4–7.9)	1		1	
Missing data	18 (1.1)	–		–	–	–	–
Surgery							
Yes	49 (3.0)	3	6.1 (1.3–16.9)	0.93 (0.28–3.04)	0.903	0.57 (0.13–2.49)	0.456
No	1555 (95.9)	102	6.6 (5.4–7.9)	1		1	
Missing data	18 (1.1)	–		–	–	–	–
Piercing							
Yes	1298 (80.0)	89	6.9 (5.5–8.4)	1.33 (0.77–2.31)	0.302	0.79 (0.42–1.96)	0.462
No	306 (18.9)	16	5.2 (3.0–8.4)	1		1	
Missing data	18 (1.1)	–		–	–	–	–
Scarification							
Yes	499 (30.8)	42	8.4 (6.1–11.2)	1.52 (1.01–2.28)	0.043	1.26 (0.80–1.96)	0.319
No	1105 (68.1)	63	5.7 (4.4–7.2)	1		1	
Missing data	18 (1.1)	–		–	–	–	–
Female genital mutilation							
Yes	1186 (73.1)	88	7.4 (6.0–9.1)	1.89 (1.11–3.22)	0.019	2.25 (1.19–4.24)	0.013
No	418 (25.8)	17	4.1 (2.4–6.4)	1		1	
Missing data	18 (1.1)	–		–	–	–	–
HIV infection							
Positive	6 (0.4)	1	16.7 (0.4–64.1)	3.01 (0.35–26)	0.317		
Negative	1556 (95.9)	97	6.2 (5.1–7.6)	1			
Missing data	60 (3.7)	–		–	–	–	–

Multivariable logistic regression by backward stepwise selection (probability of entry *p* < 0.25, probability of removal *p* < 0.3) with age group, transfusion, surgery, piercing, scarification, and female genital mutilation as forced variables. N = 1453; R^2^ = 0.033; Model *p* = 0.008.

HBsAg, Hepatitis B surface antigen; HBV, Hepatitis B virus; RDT, Rapid diagnostic test; OR, Odds ratio; aOR, Adjusted odds ratio; HIV, Human immunodeficiency virus; CI, confidence interval.

### Prevalence and factors associated with HBsAg-positive in pregnant women

**Figure 2:**
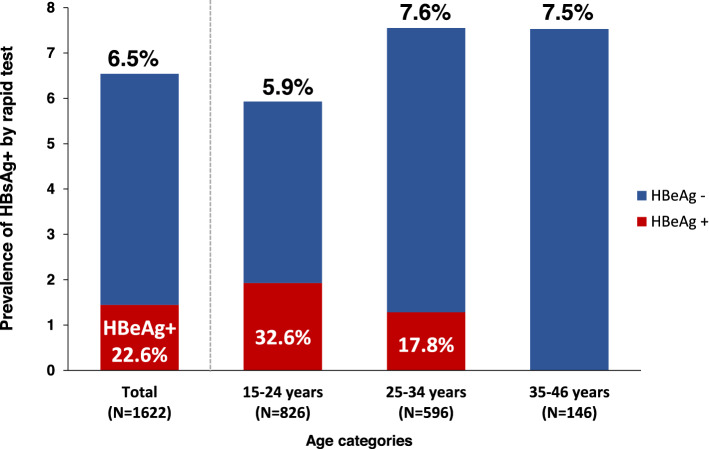
Prevalence of HBsAg and HBeAg in pregnant women in Yako, rural Burkina Faso. HBsAg was screened by rapid diagnostic test (RDT) in 1622 pregnant women visiting the study centers for antenatal care, and 106 were positive. Of the 106 HBsAg-positive by RDT, 105 DBS samples were available for serological analyses. 102 DBS samples were positive for HBsAg by CLEIA in DBS samples and underwent HBeAg measurement. The numbers on top of each graph indicate the HBsAg prevalence, while the numbers inside each bar represent the corresponding HBeAg prevalence.

The prevalence of HBsAg by rapid diagnostic test in the total sample was 6.5% (106/1622, 95% CI, 5.4–7.8%) and was slightly higher in older age groups, though not statistically significant (chi-square test *p*-value = 0.442) (Fig. 2).

Multivariable regression analysis of factors associated with HBsAg is presented in Table 1. Age 25–34 years (aOR = 2.24, 95% CI, 1.28–3.93, *p* = 0.005), having never heard of HBV (aOR = 1.63, 95%CI, 1.07–2.49, *p* = 0.022), and FGM (aOR = 2.25, 95%CI, 1.19–4.24, *p* = 0.013) were positively associated with HBV infection among pregnant women in rural Burkina Faso. On the other hand, multiparity was associated with a lower risk of HBV infection (aOR = 0.83, 95% CI, 0.47–1.45, *p* = 0.025).

### Prevalence of HBeAg, HBeAb, and distribution of viral load

Of the 106 pregnant women positive for HBsAg by rapid test, DBS samples tested positive for HBsAg by CLEIA in 102 samples and underwent HBeAg and HBeAb measurement. Overall, HBeAg prevalence was 22.6% (95% CI, 14.9–31.9%) and decreased with increasing age (chi-square test *p* = 0.040) (Fig. 2). On the other hand, HBeAb was positive in 66.7% (95% CI, 56.6–75.7%) and increased with age (chi-square *p* = 0.005).

**Figure 3:**
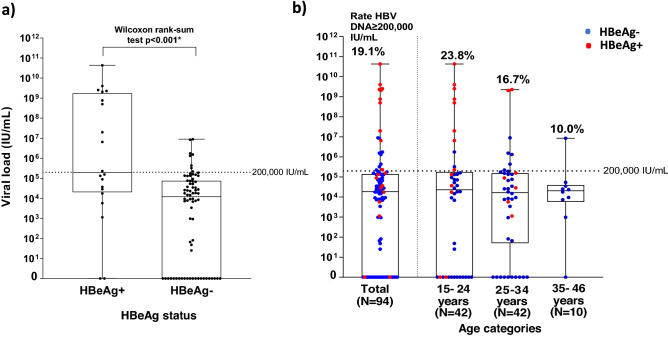
Distribution of HBV DNA by a) HBeAg and b) HBeAg and age among pregnant women in Yako, rural Burkina Faso. The red dots show HBeAg-positive cases, and the blue dots represent HBeAg-negative cases. Numbers on the top of each graph represent the percentage of pregnant women with HBV DNA ≥ 200,000 IU/mL, the recommended cut-off for antiviral prophylaxis.

HBV DNA was quantified in 94 samples. The median HBV DNA was significantly higher in HBeAg-positive pregnant women than in HBeAg-negative pregnant women (193580.0 IU/mL (IQR, 5717.461–1542185 IU/mL) vs. 12,011 IU/mL (IQR, 7804.8–133431.2 IU/mL), Wilcoxon rank-sum test *p* < 0.001) (Fig. 3a). The proportion of pregnant women with HBV DNA ≥ 200,000 IU/mL was 19.1% overall and was higher in younger age groups (Fig. 3b).

### HBV genotype distribution

HBV partial sequencing was successfully performed in 63 samples, with 60 samples sequenced for the overlapping surface-polymerase region and 3 for the surface region. Phylogenetic trees constructed to identify HBV genotypes are shown in Supplementary Figs. S1 and S2. Genotype E was predominant at 58.7% (37/63), followed by genotype A at 36.5% (23/63). Genotypes B and C were less prevalent, representing 3.2% (2/63) and 1.6% (1/63) of the HBV genotypes, respectively.

### Performance of HBeAg by using dried blood samples to identify HBV DNA ≥ 200,000 IU/mL

The performance of HBeAg to detect HBV DNA over 200,000 IU/mL (gold standard) is shown in Table 2. In the 94 HBsAg-positive cases for which viral load and HBeAg results were available, the sensitivity was 55.6% (95% CI, 30.8–78.5%) and the specificity was 86.8% (95% CI, 77.1–93.5%). By genotype, the sensitivity was 100% (95% CI, 15.8–100%) in genotype A, and 53.8% (95% CI, 25.1–80.1%) in genotype E, whereas the specificity was 66.7% (95% CI, 43.0–85.4%) in genotype A and 91.7% (95% CI, 73.0–99.0%).

**Table 2:** Performance of HBeAg by using dried blood samples to detect HBV DNA ≥ 200,000 IU/mL.

	Total samples (N = 94)	Genotype A (N = 23)	Genotype E (N = 37)
True positive, n	10	2	7
True negative, n	66	14	22
False positive, n	10	7	2
False negative, n	8	0	6
Sensitivity, % (95% CI)	55.6 (30.8–78.5)	100 (15.8–100)	53.8 (25.1–80.8)
Specificity, % (95% CI)	86.8 (77.1–93.5)	66.7 (43.0–85.4)	91.7 (73.0–99.0)
Positive predictive value, % (95% CI)	50.0 (27.2–72.8)	22.2 (2.8–60.0)	77.8 (40.0–97.2)
Negative predictive value, % (95% CI)	89.2 (79.8–95.2)	100 (76.8–100)	78.6 (59.0–91.7)
Positive likelihood ratio	4.2 (2.1–8.6)	3 (1.6–5.5)	6.5 (1.6–26.7)
Negative likelihood ratio	0.5 (0.3–0.9)	0	78.6 (59.0–91.7)
Kappa coefficient	0.4 (0.2–0.6)	0.3 (− 0.1–0.6)	0.5 (0.2–0.8)

Viral load and HBeAg were available in 94 HBsAg-positive pregnant women. HBV genotype was successfully identified in 63 samples, of which 23 belonged to genotype A and 37 to genotype E. Genotype B and C represented 2 and 1 samples, respectively, and were not assessed for the accuracy of HBeAg to detect high viral load.

## Discussion

This study revealed intermediate HBsAg prevalence (prevalence between 2 and 8%), with approximately 20% of HBsAg-positive pregnant women having high HBV DNA levels or being HBeAg-positive.

The HBsAg prevalence found among pregnant women in the Yako health district (6.5%) is similar to that of women in the nearby Nanoro health district, where the prevalence was 6.3%^16^. Such a prevalence indicates a risk of MTCT of HBV. To date, there is no routine screening for HBV infection for pregnant women in Burkina Faso, and the cost of testing and care is borne by healthcare consumers. A real-world study in urban Burkina Faso found that acceptance of HBV screening among pregnant women was high (98.2%), and up to 71.2% of counseled pregnant women underwent testing at their own expense^17^. This suggests that implementing routine HBV screening and universal health coverage for pregnant women would effectively identify HBV-infected pregnant women and prevent MTCT more adequately.

History of FGM represented the highest risk factor for HBV infection in this sample. Although prohibited, FGM is still practiced in some parts of the country, especially in rural areas where its prevalence was 80.2% in 2010^18^. FGM is usually practiced in early childhood, with poor hygiene and the reuse of infected instruments, leading to a risk of HBV transmission. Also, FGM has been reported as a risk factor for MTCT of HBV^19^. Therefore, fighting against FGM is essential to reduce the risk of HBV acquisition in young girls and MTCT transmission in their adulthood.

The prevalence of HBeAg was 22.6%, concordant with the prevalence of 18.9% (95% CI: 14.4%–23.9%) in Africa in a systematic review^20^. MTCT risk is increased when HBeAg is positive or viral load is high in pregnant women^4^. WHO recommends administering antiviral prophylaxis to HBsAg-positive pregnant women with high viral load (i.e., DNA level ≥ 200,000 IU/mL) or HBeAg-positive when viral load is unavailable. Based on the findings of this study, 19.1% of pregnant women met the criterion of high viral load, while 22.6% were HBeAg-positive. However, a number of HBeAg-positive women had low DNA levels, whereas some with high viral load were HBeAg-negative, as reported in a previous study in Burkina Faso that used rapid test for HBeAg identifcation^21^.

In this study, genotype E represented almost two-thirds of the genotypes, as found in previous reports in West Africa^16,22,23^. It is associated with a lower viral load than genotype C, which is mainly found in Asia^22^, partly explaining the difference in natural history between West African and Asian patients: Asians tend to have higher viral load and slower HBeAg loss than West Africans^24,25^.

WHO recommends using HBeAg as an alternative to viral quantification in situations where viral load is unavailable. This recommendation was based on a systematic review with meta-analysis of HBeAg to identify HBV DNA ≥ 200,000 IU/mL that found pooled sensitivity and specificity of 88.3% and 92.6%, respectively^24^. Most included studies were from Asian countries, and subgroup analysis found a lower sensitivity (82.0%) and higher specificity (96.0%) in the WHO African region. However, subgroup analysis by genotype could not be performed due to a lack of information on the genotype in most reports^24^. In our study, we found lower sensitivity and specificity for the entire cohort, with lower sensitivity in genotype E compared to genotype A. However, the interpretation of the difference in sensitivity by genotype is limited since only two pregnant women with genotype A were HBeAg-positive, and both had viral loads over 200,000 IU/mL. In Cameroon, a country where HBV genotype E is predominant, the sensitivity of HBeAg to detect high viral load in serum samples of pregnant women was reported to be 95.8%, and the specificity was 85.6%^26^. The potential influence of DBS samples on the accuracy of HBeAg detection may have resulted in the lower sensitivity observed in our study. While our previous study found good sensitivity and specificity for DBS samples to detect HBeAg^12^, literature on this matter has produced contrasting results, with some studies reporting suboptimal accuracy^27,28^. Furthermore, genotype identification by viral sequencing requires recovering a certain amount of HBV DNA from the DBS, which could be a source of spectrum bias. Therefore, the interpretation of our performance results should be approached with caution. Further studies on pregnant women, using gold standard samples and methods, are needed to clarify the accuracy of HBeAg to detect high viral load in African pregnant women, with a focus on genotypes, including genotype E, which is poorly studied and prevalent in Africa.

HBV DNA is the reference method for evaluating MTCT risk, but its availability is limited in resource-constrained countries where HBV prevalence is highest^7^. The COVID pandemic may have positively affected access to PCR platforms. Indeed, in response to COVID-19, the number of PCR platforms and trained staff has been boosted in most African countries, including Burkina Faso^29^. Therefore, this could be an opportunity to redeploy these resources to the fight against HBV infection. Another approach for resource-limited settings could be the development of point-of-care viral load tests, which could be easily used in clinical settings to facilitate the identification of pregnant women with HBV DNA ≥ 200,000 IU/mL.

Our study has some limitations. First, this study was conducted in a rural setting, and the results cannot be generalized to the whole country. In addition, questionnaire data were mainly based on the pregnant women’s self-report, which may be prone to recall or prevarication bias. Second, quantifying HBV DNA from DBS samples may have biased the actual viral load. However, this is unlikely to influence the results, as previous studies showed that the difference between DBS and paired serum samples in viral load assessment was insignificant, demonstrating the usefulness of DBS for HBV DNA quantification^30,31^. Also, a systematic review reported high sensitivity and specificity of DBS samples to perform HBV DNA quantification at 95% and 99%, respectively^32^. Third, the use of DBS samples for HBeAg measurement and genotype identification may have affected the results of HBeAg performance in identifying pregnant women with high viral load. Thus, these results should be interpreted with caution.

## Conclusion

HBsAg prevalence was intermediate in Yako, rural Burkina Faso, with a predominance of HBV genotype E. About one in five HBV-infected pregnant women had high HBV DNA levels, thereby meeting the indication for antiviral prophylaxis based on HBV viral load. Implementing routine HBV screening and effective risk assessment for MTCT for all pregnant women in Burkina Faso is essential to enable timely interventions that can reduce the incidence of HBV infection.

## Supplementary Information

Supplementary Information.

## Data Availability

The datasets used and analyzed in the current study are available from the corresponding author upon reasonable request.

## Abbreviations

aOR: Adjusted odds ratio
CI: Confident interval
CLEIA: Chemiluminescent enzyme immunoassay
DBS: Dried Blood Spot
DNA: Deoxyribonucleic acid
FGM: Female genital mutilation
HBV: Hepatitis B virus
HIV: Human immunodeficiency virus
HBsAg: Hepatitis B surface antigen
HBeAg: Hepatitis B e antigen
HBeAb: Hepatitis B e antibody
IQR: Interquartile range
MTCT: Mother-to-child transmission
PMTCT: Prevention of mother-to-child transmission
RDT: Rapid diagnostic test
WHO: World Health Organization

## References

[1] World Health Organization. Global progress report on HIV, viral hepatitis and sexually transmitted infections, 2021. Accountability for the global health sector strategies 2016–2021: actions for impact. Published online, **108** (2021).

[2] Sonderup MW, Spearman CW (2022). Global disparities in Hepatitis B elimination–a focus on Africa. Viruses.

[3] Edmunds WJ, Medley GF, Nokes DJ, Hall AJ, Whittle HC (1993). The influence of age on the development of the hepatitis B carrier state. Proc. R Soc. London Ser. B Biol. Sci..

[4] Joshi SS, Coffin CS (2020). Hepatitis B and pregnancy: Virologic and immunologic characteristics. Hepatol. Commun..

[5] World Health Organization. Interim Guidance For Country Validation of Viral Hepatitis Elimination. 1–96 (2021).

[6] World Health Organization. Global guidance on criteria and processes for validation: elimination of mother-to-child transmission of HIV, syphilis and hepatitis B virus. Published online, 86 (2021).

[7] World Health Organization. Prevention of mother-to-child transmission of Hepatitis B virus: Guidelines on antiviral prophylaxis in pregnancy. 1–58 (2020). 32833415

[8] Meda N, Tuaillon E, Kania D (2018). Hepatitis B and C virus seroprevalence, Burkina Faso: A cross-sectional study. Bull. World Health Organ..

[9] Somé EN, Kaboré J, Kaboré C (2022). Burkina Faso and the global goal of eliminating Hepatitis B virus By 2030. Adv. Public Heal. Community Trop. Med..

[10] Lingani M, Zango SH, Valéa I (2022). Low birth weight and its associated risk factors in a rural health district of Burkina Faso: A cross sectional study. BMC Pregnancy Childbirth.

[11] Nagashima S, Ko K, Yamamoto C (2021). Prevalence of total hepatitis A antibody among 5 to 7 years old children and their mothers in Cambodia. Sci. Rep..

[12] E B., Ko K., Nagashima S., *et al*. Dried blood spot‐based detection of serological profiles of hepatitis B and C infections and their prevalence in Cambodia. *GastroHep*. **3**(4): 247-253. 10.1002/ygh2.468 (2021).

[13] Matsuo J, Huy Do S, Yamamoto C (2017). Clustering infection of hepatitis B virus genotype B4 among residents in Vietnam, and its genomic characters both intra- and extra-family. PLoS ONE.

[14] Iizuka H, Ohmura K, Ishijima A (1992). Correlation between Anti-HBc titers and HBV DNA in blood units without detectable HBsAg. Vox Sang..

[15] Lingani M, Akita T, Ouoba S (2018). High prevalence of hepatitis B infections in Burkina Faso (1996–2017): A systematic review with meta-analysis of epidemiological studies. BMC Public Health.

[16] Lingani M (2020). The changing epidemiology of hepatitis b and c infections in nanoro, rural burkina faso: a random sampling survey. BMC Infect. Dis..

[17] Guingané AN, Meda N, Sombié R (2016). Prevention of mother-to-child transmission of hepatitis B in the Urban district health baskuy Burkina Faso. Open J. Gastroenterol..

[18] Chikhungu LC, Madise NJ (2015). Trends and protective factors of female genital mutilation in Burkina Faso: 1999 to 2010. Int. J. Equity Health.

[19] Sangaré L, Sombié R, Combasséré AW (2009). Antenatal transmission of hepatitis B virus in an area of HIV moderate prevalence Burkina Faso. Bull. Soc. Pathol. Exot..

[20] Bigna JJ, Kenne AM, Hamroun A (2019). Gender development and hepatitis B and C infections among pregnant women in Africa: A systematic review and meta-analysis. Infect. Dis. Poverty..

[21] Guingané AN, Kaboré R, Shimakawa Y (2022). Screening for Hepatitis B in partners and children of women positive for surface antigen Burkina Faso. Bull. World Health Organ..

[22] Wongjarupong N, Yonli AT, Nagalo BM (2020). Characteristics of patients with chronic Hepatitis B virus infection with Genotype E predominance in Burkina Faso. Hepatol. Commun..

[23] Ingasia LAO, Wose Kinge C, Kramvis A (2021). Genotype E: The neglected genotype of hepatitis B virus. World J. Hepatol..

[24] Boucheron P, Lu Y, Yoshida K (2021). Accuracy of HBeAg to identify pregnant women at risk of transmitting hepatitis B virus to their neonates: A systematic review and meta-analysis. Lancet Infect. Dis..

[25] Shimakawa Y, Lemoine M, Njai HF (2016). Natural history of chronic HBV infection in West Africa: A longitudinal population-based study from The Gambia. Gut.

[26] Shimakawa Y, Veillon P, Birguel J (2022). Residual risk of mother-to-child transmission of hepatitis B virus infection despite timely birth-dose vaccination in Cameroon (ANRS 12303): A single-centre, longitudinal observational study. Lancet Glob. Heal..

[27] Kikuchi M, Lindstrom P, Tejada-Strop A, Mixson-Hayden T, Kamili S, Sawabe M (2022). Dried blood spot is the feasible matrix for detection of some but not all hepatitis B virus markers of infection. BMC Res. Notes.

[28] Jackson K, Holgate T, Tekoaua R, Nicholson S, Littlejohn M, Locarnini S (2022). Evaluation of dried blood spots for hepatitis B and D serology and nucleic acid testing. J. Med. Virol..

[29] Talisuna A, Iwu C, Okeibunor J (2022). Assessment of COVID-19 pandemic responses in African countries: Thematic synthesis of WHO intra-action review reports. BMJ Open.

[30] Jardi R, Rodriguez-Frias F, Buti M (2004). Usefulness of dried samples for quantification and molecular characterization of HBV-DNA. Hepatology.

[31] Stene-Johansen K, Yaqoob N, Overbo J (2016). Dry blood spots a reliable method for measurement of Hepatitis B viral load in resource-limited settings. PLoS ONE.

[32] Lange B, Roberts T, Cohn J (2017). Diagnostic accuracy of detection and quantification of HBV-DNA and HCV-RNA using dried blood spot (DBS) samples–a systematic review and meta-analysis. BMC Infect. Dis..

