# Supplementary material for: Intermediate hepatitis B virus infection prevalence among 1622 pregnant women in rural Burkina Faso and implications for mother-to-child transmission
Source: Sci Rep. 2023 Apr 14;13:6115. doi: 10.1038/s41598-023-32766-3 (PMC10103033; doi:10.1038/s41598-023-32766-3)
Supplement: Supplementary file 1 — Supplementary Information. [file 41598_2023_32766_MOESM1_ESM.pdf]

Supplementary Information

Intermediate hepatitis B virus infection prevalence among 1622 pregnant women in rural Burkina Faso and implications for mother-to-child transmission

Serge Ouoba, Ko Ko, Moussa Lingani, Shintaro Nagashima, Alice Guingane, Bunthen E, Md Razeen Ashraf Hussain, Aya Sugiyama, Tomoyuki Akita, Masayuki Ohisa, Moussa Abdel Sanou, Ousmane Traore, Job Wilfried Nassa, Maimouna Sanou, Kazuaki Takahashi, Halidou Tinto, Junko Tanaka

Supplementary Table S1. Primers used for nested PCR and partial genome sequencing

a) Nested PCR

Target region	Primer name	Stage polarity	Nucleotide position	Nucleotide sequence (5'-3')	Reference
Surface-polymerase (SP) region	S1	PCR 1st Sense	1414–1434	ACGTCCTTTGTTTACGTCCCG	13
	S2	PCR 1st Sense	1436–1456	CGGCGCTGAATCCCGCGGACG	13
	AS1	PCR 2nd Sense	2130–2110	TCCAAATTACTTCCCACCCAG	13
	AS2	PCR 2nd Sense	2160–2140	CTGACTACTAATTCCCTGGAT	13
	S3	PCR 1st Antisense	1489–1508	CCGCTTCTCCGTCTGCCGTA	13
	S4	PCR 1st Antisense	1527–1547	CACCTCTCTTTACGCGGACTC	13
	AS3	PCR 2nd Antisense	2185–2165	TAGGCCCATATTAACATTGAC	13
	AS4	PCR 2nd Antisense	2098–2078	CATCAACTCACCCCAACACAG	13
Surface (S) region	S1-1	PCR 1st Sense	192-211	TCGTGTTACAGGCGGGGTTT	14
	S1-2	PCR 2nd Sense	455-474	CGAACCACTGAACAAATGGC	14
	S2-1	PCR 1st Antisense	685-704	CAAGGTATGTTGCCCGTTTG	14
	S2-2	PCR 2nd Antisense	668-687	GGCACTAGTAAACTGAGCCA	14

b) Genome sequencing

Target region	Primer name	Nucleotide sequence (5'-3')	Length	Target nucleotide position	Reference
**SP region**	AS4	CATCAACTCACCCCAACACAG	458 bp	475-933	13
**S region**	S2-2	GGCACTAGTAAACTGAGCCA	232 bp	455-687	14

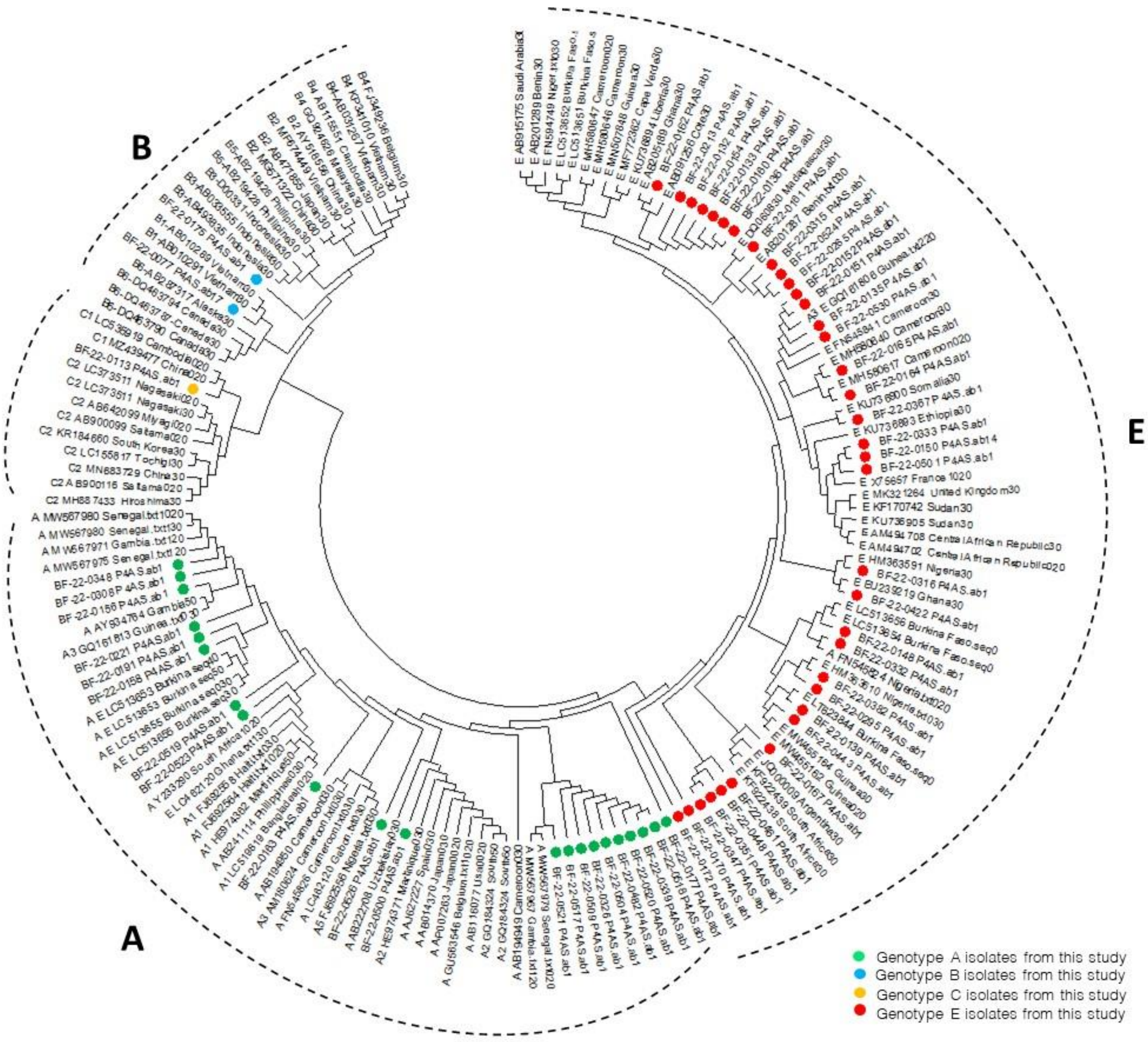

Supplementary Figure S1. Phylogenetic tree of the overlapping surface-polymerase region of HBV genome

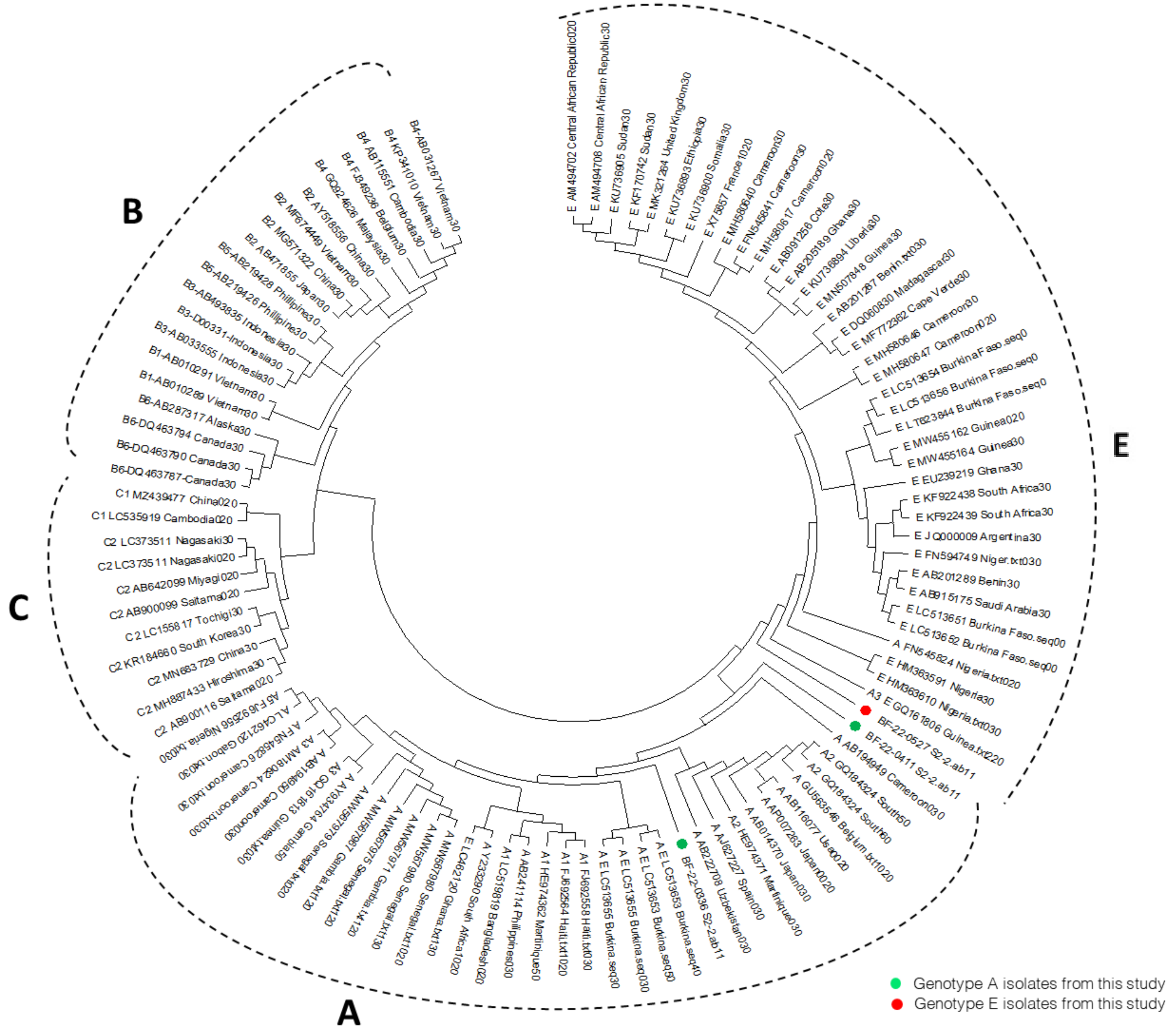

Supplementary Figure S2. Phylogenetic tree of the surface region of HBV genome

Phylogenetic trees were constructed by the neighbor-joining method with the Molecular Evolutionary Genetics Analysis software version 10 (Pennsylvania State University, PA, USA) using 103 reference strains retrieved from GenBank.
